# Harnessing genomics for fast development and commercial introduction of high-resistant tomato varieties to Tomato Brown Rugose Fruit Virus

**DOI:** 10.1270/jsbbs.25047

**Published:** 2026-02-14

**Authors:** Walter Verweij, Sergio de la Fuente van Bentem, Marieke Ykema, Fréderic Perefarres, Nejra Solo, Albert Grit, Louison Liard, Bas ter Riet, Martijn van Stee, Karin Posthuma, Gert-Jan de Boer, Jean-François Thomin, Rob J. Dekker, Antoine Janssen, Jeroen Rouppe van der Voort, Kees Könst

**Affiliations:** 1 Enza Zaden Research & Development B.V., Haling 1E, 1602 DB, Enkhuizen, the Netherlands; 2 RNA Biology & Applied Bioinformatics research group, Swammerdam Institute for Life Sciences, Faculty of Science, University of Amsterdam, Amsterdam, the Netherlands; 3 Keygene N.V., Agro Business Park 90, Wageningen, the Netherlands

**Keywords:** ToBRFV, *Solanum lycopersicum*, plant breeding, genetic mapping, next-generation sequencing, gene cloning, R gene based resistance

## Abstract

Tomato plants are susceptible to a wide range of viruses, including those belonging to the genus *Tobamovirus*, which pose enormous threats to tomato cultivation worldwide. This article reviews a genomics-based breeding strategy applied for the rapid discovery and introgression of the newly identified *HREZ* resistance gene, which provides high-resistance to the recently emerged *Tobamovirus fructirugosum* (Tomato Brown Rugose Fruit Virus (ToBRFV)). From the identification of a novel viral tomato pathogen in 2015, an applied breeding strategy allowed us to launch a series of 18 tomato varieties within a time span of seven years.

## Introduction

The genus *Tobamovirus* includes several highly infectious species that cause serious diseases in tomato (*Solanum lycopersicum* L.) with high economic impact. In addition to practical hygiene measures, such as strict control and checks on seed and plant health, an effective method of controlling viral diseases in tomato is by utilizing major resistance genes (R genes). So far, tomato breeders have developed resistant varieties that harbor specific *Tobamovirus* resistance genes such as *Tm-1*, *Tm-2* and *Tm-2^2^* (Hall 2004, Lanfermeijer *et al.* 2005, Pelham 1966). The *Tm-2^2^* gene is of particular importance to the tomato industry as it has provided durable resistance over the last 50 years (Ishibashi *et al.* 2014, Lanfermeijer *et al.* 2004) to a range of *Tobamovirus* species.

Early 2015, we received samples from diseased tomato plants carrying the *Tm-2^2^* resistance gene but showing the mosaic patterns on leaves accompanied occasionally by narrowing of leaves and yellow spotted fruits that are typical for *Tobamovirus* infections on tomatoes. Diagnostics research showed that these symptoms were caused by a new *Tobamovirus*, later called Tomato Brown Rugose Fruit Virus (ToBRFV) (Luria *et al.* 2017, Salem *et al.* 2016) and in the 2024 taxonomic review renamed as *Tobamovirus fructirugosum*. In the following years, this new *Tobamovirus* caused a pandemic in the tomato industry, spreading widely to officially reported in 46 countries (EPPO 2025) and affecting almost all countries where tomato fruits are produced in greenhouses and open field, resulting in estimated losses of hundreds of millions of US Dollars on an annual basis (Salem *et al.* 2023, Turina and Salem 2022).

Here we review a genomics-based breeding strategy that resulted in the development of 18 new resistant tomato (*S. lycopersicum*) varieties within a timespan of seven years. Upon first identification of ToBRFV through an internal diagnostic analysis of samples in 2015, we initiated a major screening process of plant genetic resources, including wild tomato relatives, to identify new resistant sources. Tomato wild relatives are tremendous sources of genetic diversity and can be used to identify new resistances and tolerance to biotic and abiotic challenges (Tirnaz *et al.* 2022). However, the use of crop wild relatives in modern breeding presents various obstacles and the introgression of a resistance gene from a wild relative into a modern tomato variety can be laborious and time-consuming. In most cases, a timeline for development of a new tomato variety exceeds a decade (Menda *et al.* 2014, Wang *et al.* 2024).

This paper describes the various steps during the process of creating our first series of tomato varieties showing high resistance to ToBRFV. The application of genomics techniques was the decisive factor in enabling our breeding team to be the first in the market by introduction of high-resistant ToBRFV tomato varieties.

## Time-line of the development of ToBRFV-resistant tomato varieties

**Fig. 1.**
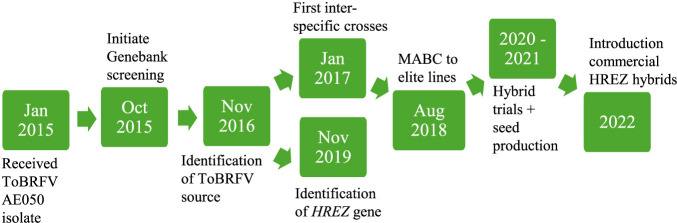
Timeline of the 7-year process starting with the receipt of a sample containing the ToBRFV AE050 isolate (Jan 2015) until the commercial introduction of the first HREZ tomato varieties (2022).

In this section, we describe our breeding process in chronological order. A schematic overview is provided in Fig. 1.

### January 2015–July 2015: characterization of diagnostic samples

Samples from symptomatic tomato plants carrying *Tm-2^2^* gene were collected from production fields in Saudi Arabia and Jordan on January 26th and June 3rd 2015, respectively. Based on visual observation of plants and samples, i.e. necrotic lesions on leaves and strong rugose symptoms on fruits, we hypothesized that the disease was caused by a potentially novel *Tobamovirus* spp. ToMV ELISA confirmed that *Tobamovirus* spp. was present in these samples. Under quarantine conditions, we established a test isolate from these samples, which was further subjected to next-generation sequencing. This first sample received is referred to as isolate AE050.

Tomato (*S. lycopersicum*) and *Nicotiana benthamiana* seedlings were rub-inoculated and symptoms started to appear after two weeks, showing the typical characteristics of a *Tobamovirus* infection. Small-RNA-sequencing was used to discover virus-derived siRNAs in total RNA isolated from the tomato leaf samples (Locati *et al.* 2015). After *in-silico* removal of all tomato-derived sequences, we generated a contiguous ~6.5 kb consensus nucleotide sequence, supported by 603,000 reads, with an average coverage of ~2,200X to generate a *de novo* assembly. To corroborate the viral origin of this *de novo* assembly, the filtered reads were mapped back to this contig with default miRNA alignment settings, which effectively also identifies siRNAs. Of the 1,322,645 reads, 656,426 (49.63%) aligned covering the complete contig sequence. The final consensus viral genome sequence was derived from this alignment.

This consensus nucleotide sequence showed 82% similarity to Tobacco Mosaic Virus, classifying it clearly as a different and novel species as at the time the NCBI nucleotide database did not include a sequence more than 90% similar to this consensus sequence. The next-generation sequencing result was substantiated by a publication in November 2015 (Salem *et al.* 2016) reporting on a new *Tobamovirus* spp. infecting tomato crops in Jordan called tomato brown rugose fruit virus (ToBRFV).

### October 2015–November 2016: screening wild tomato accessions

Young plants from a large internal gene bank collection of wild tomato species, including *S. pennellii*, *S. peruvianum*, *S. chilense*, *S. habrochaites*, *S. pimpinellifolium*, *S. neorickii*, *S. corneliomulleri*, *S. chmielewskii*, *S. cheesmaniae* and *S. galapagense*, were subjected to ToBRFV AE050 inoculation and disease progression was monitored. In total more than 800 accessions were screened using 12 plants per accession. Plants that did not show symptoms were selected, re-inoculated, further evaluated and subjected to ELISA.

**Fig. 2.**
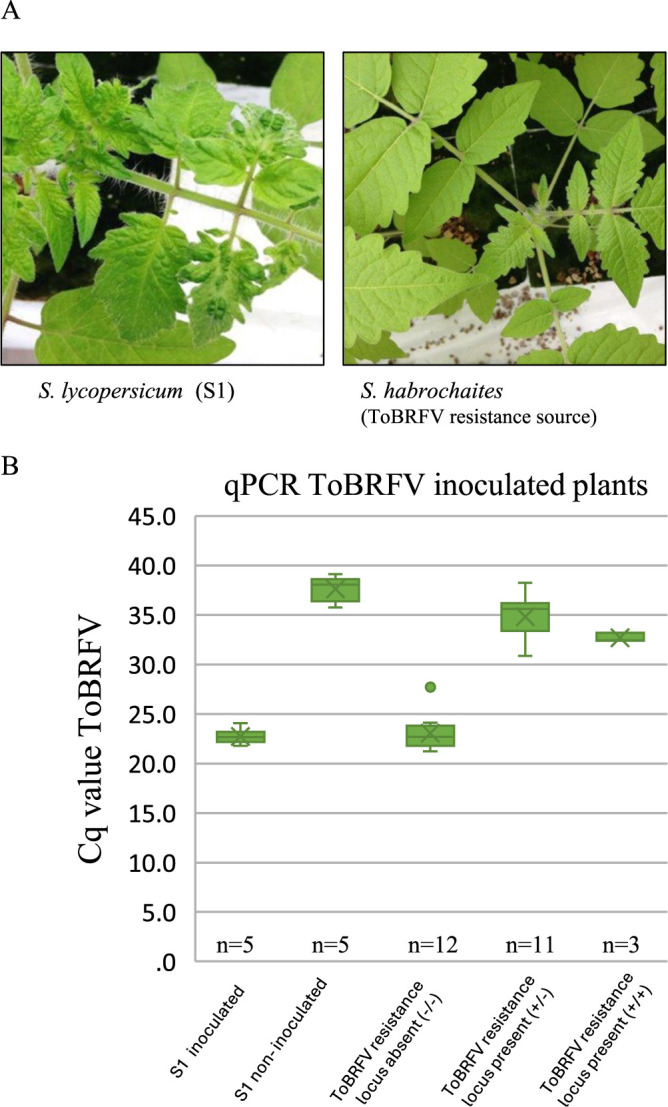
(A) Phenotypes (15 weeks after ToBRFV inoculation) of susceptible *S. lycopersicum* (S1) plants and the identified ToBRFV resistant *S. habrochaites* source. (B) qPCR detection of six-week-old ToBRFV inoculated and non-inoculated susceptible *S. lycopersicum* (S1) plants and ToBRFV resistant *S. habrochaites*. S1, ToBRFV inoculated (n = 5), S1 non-inoculated (n = 5), ToBRFV resistant locus absent (–/–) (n = 12), ToBRFV locus present (+/–) (n = 11), ToBRFV locus present (+/+) (n = 3). The Cq values or quantification cycle values represent the number of PCR cycles needed for the signal to become detectable and is a measure of the amount of virus particles present in the sample (Supplemental Table 6).

This extensive screening resulted in several *S. pimpinellifolium* and *S. habrochaites* accessions that remained free from virus symptoms during each plant developmental stage. ELISA and qPCR were used to detect the presence of virus particles and showed that only LYC4943, a *S. habrochaites* accession from Peru, remained virus-free for more than 15 weeks after inoculation (Fig. 2) (Ykema *et al.* 2020).

### November 2016–March 2018: fine-mapping ToBRFV resistance locus

Soon after the resistant source was validated, nine plants from the resistant accession were crossed with *S. lycopersicum* line S1 to create nine F_1_ families. Four F_1_ families germinated and were tested in the bioassay revealing a dominant genetic factor as all tested F_1_ plants remained free of symptoms and free of virus (June 2017). Two backcross (BC_1_) populations were generated by crossing F_1_ plant (LYC4943 plant 3 (#90479-3) x S1) with two ToBRFV susceptible *S. lycopersicum* lines (S1 and S2) (Jan 2017, Fig. 1). In March 2018, a total of 782 plants (298 plants of ((S1 x #90479-3) x S1) and 484 plants of ((S2 x #90479-3) x S2) were genotyped with nine randomly distributed SNP markers (Supplemental Table 1), that distinguish *S. habrochaites* from *S. lycopersicum*, to ensure that the BC_1_ population did not contain self-pollinated progenies. In addition, the plants were inoculated with the ToBRFV isolate AE050 and two to three weeks after inoculation, a visual phenotyping scoring was done. A clear 3:1 segregation was observed between resistant and susceptible plants pointing towards a single dominant gene. Interestingly, the SNP marker on chromosome 8 (position 2673609 on the SL2.40 reference genome) showed a linkage to the ToBRFV resistant phenotype. Further studies focused on chromosome 8, using a set of 21 markers (Supplemental Table 2) to genotype 92 individual plants. This resulted in a reduced region of interest at chromosome 8 between M8 and M14, which are positioned on 53120868 and 57038520 of the Heinz v2.40 reference genome, respectively.

**Fig. 3.**
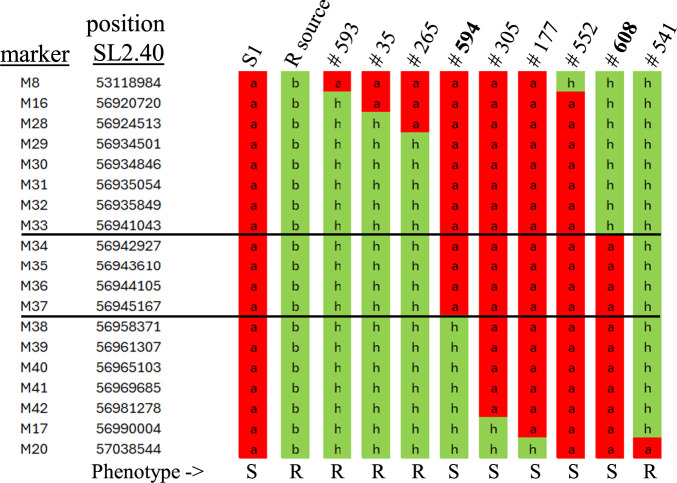
Genotyping and phenotyping results of recombinant plants between M8 and M20 on chromosome 8. The physical positions are on the Heinz reference genome V2.40. S1 is the susceptible control, the R source is the original *S. habrochaites* source that was identified in the gene bank screen. Marker score “a” indicates absence of the ToBRFV resistance locus, a marker score “b” indicates homozygous presence of the ToBRFV resistance locus, a marker score “h” means the ToBRFV resistance locus in heterozygous state. The region between the black line indicates the fine mapped ToBRFV locus.

**Fig. 4.**
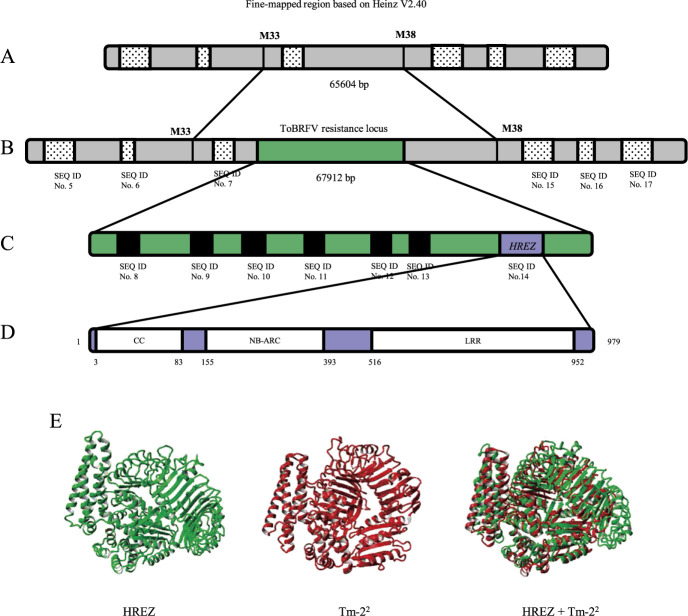
(A) Representation of the initially mapped ToBRFV resistance locus on chromosome 8 based on the SL2.40 reference. The shown fragment is 65604 bp in length and contains 6 annotated genes (dashed). (B) the fine-mapped ToBRFV resistance locus based on the *de novo* assembly of the homozygous #90479-3 plant and consists of 133516 bp. The additional genomic fragment (in green) present in the ToBRFV resistant source consists of 67912 bp. (C) Representation of the 7 additional genes (in black) present in the genomic fragment deriving from the ToBRFV resistant source. SEQ ID No. 5, SEQ ID No. 6, SEQ ID No. 7, SEQ ID No. 8, SEQ ID No. 9, SEQ ID No. 10, SEQ ID No. 11 encode a CC-NBS-LRR resistance gene, SEQ ID No. 12 and SEQ ID No. 13 encode Receptor kinase-like proteins. SEQ ID No. 15 and SEQ ID No. 16 encode a Formin 2B protein while SEQ ID No. 17 encode an unknown protein. SEQ ID No. 14 represents the *HREZ* CC-NBS-LRR resistance gene. (D) A schematic representation of the HREZ protein with the CC, NB-ARC and LRR domains which are predicted based on the NCBI CD-Search. (E) Prediction of the HREZ (green) and Tm-2^2^ (red) three-dimensional protein structure. The right image depicts the two proteins superimposed. The protein structures were obtained by modelling via Boltz-2 (https://boltz.bio/boltz2) and were structurally aligned with MUSTANG by Yasara Software.

For fine-mapping, all 782 BC_1_ plants from the two crosses, were genotyped with the flanking markers M8 and M20 and two additional markers (M12 and M13) to identify recombinant plants. In total 21 plants, recombining in the region of interest, were recovered. They were further phenotyped with isolate AE050 and genotyped with 19 additional SNP markers (M8–M20; Fig. 3 and Supplemental Table 3) in order to further reduce the ToBRFV resistance locus. Two recombinant plants provided evidence that the ToBRFV resistance locus is located between M33 and M38 on chromosome 8, which correlates to a region of 17328 bp based on the Heinz v2.40 reference genome (Fig. 3). Remarkably, based on *in-silico* prediction analysis (*S. lycopersicum* Heinz v2.40), this locus is comprised of one gene showing high similarity to *Solyc08g075630* encoding a classical nucleotide binding site, leucine rich repeat protein containing a coiled-coil domain (CC-NBS-LRR, Kourelis and van der Hoorn 2018) (SEQ ID No. 7, Fig. 4A). Since the Heinz variety is not resistant to ToBRFV, it was considered highly unlikely that the *Solyc08g075630* gene conferred resistance to ToBRFV.

### July 2017–July 2019: characterization of the ToBRFV resistance locus

The presence of additional and unexpected peaks in the high-resolution melting curve analyses of some of the chromosome 8 SNP markers made us conclude that an additional genomic region must be present in the ToBRFV resistance donor. To elucidate the DNA sequence of the fine-mapped ToBRFV resistance locus in the original *S. habrochaites* source, genomic DNA was isolated from a resistant plant (*S. lycopersicum*, plant #90479-3) in which the ToBRFV resistance locus is present in homozygous configuration. High quality sequencing libraries were generated to sequence in the Oxford Nanopore (ONT) system. In total ~117 Gbp of DNA sequence was obtained, which translates to a ~123X coverage of the assumed genome size of the tomato genome (950 Mbp). The largest reads that together resulted in a 30X coverage were selected, having a minimum read length of 33842 bp.

Short-read Illumina data were obtained to carry out quality control of the obtained ONT sequences. To measure gene completeness in the *de novo* assembly, the Benchmarking Universal Single-Copy Orthologs method was used (BUSCO; Simão *et al.* 2015, Waterhouse *et al.* 2018). BUSCO provides quantitative measures for the assessment of genome assembly, gene set, and transcriptome completeness, based on near-universal single-copy orthologs selected from Ortho DB v9. Our analysis resulted in the detection of 92% (1331 out of 1440) BUSCO genes in the Illumina data polished assembly whereas without polishing, only 61% of the BUSCO genes were found. These findings provide strong evidence that Illumina short-read data contributes to generating a more complete genome assembly (Table 1).

Because the ToBRFV resistant locus was mapped to chromosome 8, we could specifically select the contig in the *S. habrochaites* genome assembly that is homologous to the corresponding Heinz v2.40 region. From all the 1243 contigs, only Contig0017 showed high similarity with fine-mapped region and to our surprise, this Contig0017 was approximately 68 Kbp larger than the Heinz v2.40 region (85240 bp vs. 17328 bp, respectively). It was therefore considered that one or more genes are located within this region, indicated in Fig. 4A as “ToBRFV resistance locus”, is providing the ToBRFV resistance (Fig. 4B).

Blasting the DNA sequences derived from the ToBRFV resistance locus against the database of the National Center for Biotechnology Information (NCBI), resulted in seven genes of which five encode NBS-LRR resistance proteins (SEQ ID No. 8, SEQ ID No. 9, SEQ ID No. 10, SEQ ID No. 11 and SEQ ID No. 14) and two genes have homology with receptor-like kinases (SEQ ID No. 12 and SEQ ID No. 13) as depicted in Fig. 4C and Supplemental Table 4.

To further resolve the ToBRFV resistance locus and identify the gene providing ToBRFV resistance, we sequenced the resistant plant #90479-3 and analyzed the results using the Iso-Seq analysis application (Pacific Biosciences of California, PacBio). This resulted in only one candidate resistance gene transcript located in a region between markers M33 and M38. This candidate ToBRFV resistance gene is represented by SEQ ID No. 14 encoding a CC-NBS-LRR protein and is hereinafter referred to as the *HREZ* (*High Resistance Enza Zaden*) gene (Ykema *et al.* 2020). A conserved domain search of the HREZ protein shows the Coiled-coil, NB-ARC and Leucine-rich repeats (Fig. 4D) which are typical for NBS-LRR proteins.

### July 2019–Nov 2019: validation of the ToBRFV resistance gene by VIGS

To confirm that the *HREZ* gene (SEQ ID No. 14) was indeed the gene conferring resistance to ToBRFV, a Virus Induced Gene Silencing (VIGS) analysis was performed using Tobacco Rattle Virus (TRV)-derived VIGS vectors according to the method of Huang (Huang *et al.* 2012). Based on the DNA sequence similarity between all genes in the fine-mapped region, we designed two VIGS constructs (Supplemental Table 5) that specifically silence the NBS-LRR resistance genes. Construct ToBRFV-01a specifically silenced gene *Solyc08g075630* and the *HREZ* gene. Construct ToBRFV-01b targeted the other NBS LRR genes at the ToBRFV resistance locus as depicted in Fig. 4C.

**Fig. 5.**
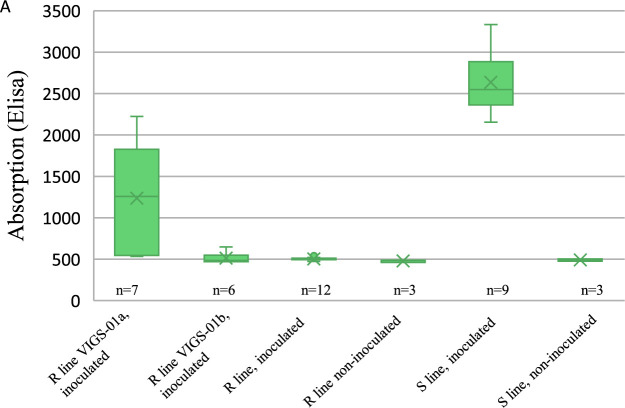
Results of the VIGS experiment using a ToBRFV resistant line (R line 15322-04) and S line control plants (S1). The resistant plants were VIGS silenced with constructs VIGS-01a and VIGS-01b. ToBRFV measurement was done using ELISA. R line with VIGS-01a construct (n = 7), ToBRFV inoculated, R line with VIGS-01b construct ToBRFV inoculated (n = 6), R line, ToBRFV inoculated (n = 12), R line, non-inoculated (n = 3), S line, ToBRFV inoculated (n = 9), S line, non-inoculated (n = 3).

Two different genotypes were used in the VIGS experiment: a homozygous ToBRFV resistant line (#15322-04) as well as the susceptible control line (S1). As expected, all plants of the susceptible control line were found susceptible to ToBRFV. Silencing of the candidate *HREZ* gene via VIGS using construct VIGS-01a resulted in all plants of the ToBRFV resistant line #15322-04 to show symptoms upon inoculation with ToBRFV isolate AE050. Silencing using the VIGS-01b construct did not result in any symptoms on the tested plants. Based on the results of the fine-mapping and VIGS experiments, it can be concluded that the newly identified *HREZ* gene confers resistance to ToBRFV (Fig. 5).

### August 2018–Spring 2022: introgression of the ToBRFV resistance gene into elite breeding material

One self-pollinated BC_2_ plant ((S1 x #90479-3) x S1) and a plant homozygous for the *HREZ* gene were selected as a genitor for marker assisted backcrossing (MABC). This approach was used to create ToBRFV resistant parental lines to produce hybrids for six different crop segments. This BC_2_S_1_ genitor was selected based on the size of the introgression segment as well as plant phenotype. In general, for each parental combination, approximately 100 segregating markers were selected to cover the tomato genome and two HREZ flanking markers were used. For the BC_1_ generation, a total of six BC_1_ plants were selected based on the highest similarity with the recurrent parent genome. These six BC_1_ plants were used to generate a second backcross generation. For each selected BC_1_, six BC_2_ plants were selected based on the highest percentage recurrent parent genome, resulting in 36 BC_2_ plants in total. The subsequent 36 BC_2_ plants were self-pollinated and BC_2_S_1_ plants were selected which harbored the *HREZ* gene in homozygous configuration. These homozygous BC_2_S_1_ plants were compared next to the original parental line and evaluated based on plant phenotype. On average, six BC_2_S_1_ plants were selected for making test hybrids per commercial segment. Each of the test hybrids were compared to the original hybrids for final selection of the optimal parent combination. Once the optimal parent combination was defined, commercial seed productions were commenced resulting in commercial seed batches within a period of six months. This procedure allowed us to introduce a total of 18 commercial hybrids, covering 6 commercial segments in spring 2022 (Table 2). As of the writing of this manuscript (August 2025), we have converted 93% of our product portfolio into a total of 67 HREZ varieties covering 15 commercial segments.

## Conclusion and Perspectives

### Genomics mediated development of ToBRFV-resistant tomato varieties

This review highlights the use of genomics tools and technologies for identification of new viral pathogens and rapid introgression of genetic resistance genes (Luria *et al.* 2017, Salem *et al.* 2016). We describe a strategy that combined the latest molecular technologies in order to characterize a new *Tobamovirus* spp., to identify a highly resistant tomato source, and to map and validate the responsible resistance gene (*HREZ* gene). This was followed by a rapid introduction of the resistance in a series of highly resistant elite tomato varieties.

Screening the entire collection and identifying the most resistant source took over a year and once a putative resistant wild species was identified, we applied genomics tools at various stages of the breeding process to gain maximum speed and precision in the breeding process.

### Characterization of a new viral species

For characterization of the causal viral species, we applied a novel method to discover new plant viruses. This method combined the analysis of plant RNA with RNA-seq and small RNA-seq of host generated siRNAs. The RNA-seq approach involves analyzing plant RNA using RNA-seq and bioinformatics to assemble reads into contigs, which are then compared to virus databases to detect similarity to known virus sequences. However, challenges of this method are a high background of host RNA and the inability to detect past virus infections due to the absence of viral RNA. On the other hand, the siRNA approach investigates the virus-related siRNA defense response of host plants (Vaucheret and Voinnet 2024). This response, which can cover the entire length of RNA viruses, can help to reconstruct the complete virus RNA sequence from host-generated siRNAs (Wu *et al.* 2010). An advantage of this method is that it can provide information even on past virus infections, as the siRNA response may persist after the virus is defeated. However, it struggles with distinguishing multiple virus variant sequences due to the short length (21 nucleotides) of siRNAs. Combining small RNA-seq and RNA-seq in virus discovery experiments leveraged the strengths of both approaches and provided a more comprehensive detection method.

### A gene cloning strategy was essential to minimizing the occurrence of linkage drag and to understanding the resistance mechanism

We set out for a fine mapping and gene cloning strategy to identify the gene responsible for ToBRFV resistance. Fine mapping resulted in a set of DNA markers closely linked to the target gene, which allowed selection of plants with a short introgression segment. Introgression of wild-species alleles into cultivated crop species is often impeded by the occurrence of deleterious alleles, closely linked to the target gene. To remove these unwanted alleles, many rounds of backcrossing using large segregating populations may be required. We minimized the risk of the occurrence of linkage drag by crossing with two recurrent parent lines and using large backcross populations at the start of the breeding process. By means of this method, we could reduce the size of the introgression segment to approximately 68 Kbp where, to the best of our knowledge, no deleterious alleles are located.

A second reason for applying a gene cloning strategy is to understand the mechanism of the *HREZ* gene-based resistance. The *HREZ* gene is classified as a so-called R gene which encodes a CC-NBS-LRR resistance protein. Plant disease resistance based on dominant R genes often results in “high resistance” (HR) plant varieties: plant varieties that highly restrict the growth and/or development of the specified pest and/or the damage it causes under normal pest pressure when compared to susceptible varieties (De Ronde *et al.* 2014, International Seed Federation 2023). HR based resistance is the preferred type of host-plant resistance to a pathogen as it results in the restriction of growth and development of the pathogen. *HREZ* mediated ToBRFV resistance is defined as HR because *HREZ* varieties consistently show significant higher Cq values, indicating very low amounts of virus particles, compared to (1) Intermediate Resistance (IR) showing slightly lower Cq values, or (2) susceptible plants, which have the lowest Cq values (Vos *et al.* 2022). In practice, this implies that although varieties with intermediate resistance to ToBRFV may perform well under grower conditions, they continue to build up virus load in the crop as has been shown by Ghijselings and coworkers (Ghijselings *et al.* 2023).

### Simultaneous Marker-assisted Backcross (MABC) projects resulted in a portfolio of HREZ varieties

The global presence of ToBRFV in almost all market segments prompted us to initiate a broad MABC program focusing on the fast introduction of the *HREZ* gene into a wide range of varieties. This implied that selections carried out on the backcross progenies generated from BC_2_S_1_ plant #90479-3 were exclusively executed based on DNA markers. This allowed us to select from the initial #90479-3 cross until the production of commercial seed lots for a total 18 varieties in less than four years (Table 2). A drawback of this fast-acting approach is that we erroneously incorporated, in some breeding lines, a genetic factor resulting in an aberrant phenotype, which is only visible when seedlings are exposed at temperatures above 28°C. When heat stress is present, the aberrant phenotype was observed at seedling stage from the moment the plants develop first true leaves until adult plant stage. The aberrant phenotype was seen as deformed and thinned stiff leaves that can curl, also called “shoestring plants”. Plants appeared weaker and were delayed in development. In the seedling stage in particular, the true leaves were stunted. In adult plants, the aberrant phenotype was associated with a poor fruit set. As soon as this aberrant phenotype appeared in our selections, we suspected linkage drag at the introgression segment at chromosome 8. However, after executing an extensive genetic mapping it appeared that this aberrant phenotype (*AP-1*) locus co-segregated in some of our selections and was located at the top of chromosome 9 (Liard *et al.* 2024). Because the mapping experiment provided closely linked markers to the *AP-1* locus, it was relatively easy to cross out the *AP-1* locus from further selections.

### HREZ breaking ToBRFV variants are found but appear to have a reduced fitness

The CC-NBS-LRR structure of the HREZ protein (Fig. 4D) suggests that it binds to an epitope on one of the four viral proteins which are encoded at the ToBRFV genome. Indeed, variants of the ToBRFV virus have been found (Botermans *et al.* 2023, Jewehan *et al.* 2022a, Zisi *et al.* 2024). Most of these variants show at least one mutation in the movement protein (MP). This suggests that the MP triggers an active resistance response similar to the mode of action of the *Tm-2^2^* gene in tomatoes resistant to ToMV and TMV. (Pfitzner 2006). Interestingly, ToBRFV isolates that contain amino acid variation in the movement protein show a decreased fitness compared to isolate AE050. When we multiply the variant strains on *HREZ* containing plants, we observe a decrease of 50% in ToBRFV virus presence. In subsequent multiplication on a second set of *HREZ* plants, these strains are no longer virulent. This suggests that, under natural conditions, the incidence of ToBRFV isolates capable of breaking the *HREZ* gene can be controlled by removing infected plants, like what has been observed with variant strains of ToMV which were able to multiply on *Tm-2^2^* resistant tomatoes.

### The HREZ gene is temperature sensitive

Another feature of the *HREZ* gene is that similar to several other CC-NBS-LRR genes, its effectiveness is impeded at temperatures above 30°C (Jewehan *et al.* 2022b). Especially when young HREZ-containing tomato plants are grown under high ToBRFV pressure, high temperatures should be prevented to avoid virus accumulation. Further studies in our company have shown that the negative effects of high temperatures can be mitigated by combining the *HREZ* gene with the *Tm-1* gene at chromosome 2 (De la Fuente van Bentem *et al.* 2024, Ishibashi *et al.* 2007) or an introgression segment at chromosome 11 (De la Fuente van Bentem *et al.* 2024).

### Adoption of an HREZ mediated ToBRFV resistance strategy may be durable if it becomes a market standard

It should be noted that the use of specific R genes is not a silver bullet and must be combined with the correct application of strict hygiene measures, seed health testing and use of virus-resistant rootstocks (Chanda *et al.* 2021, Davino *et al.* 2020, Samarah *et al.* 2021, Spanò *et al.* 2020). In retrospect, the success of the use of the *Tm-2^2^* gene against TMV and ToMV can only be explained by the fact that at the emergence of *Tm-2* breaking ToMV strains, the tomato seed industry collectively started to use the *Tm-2^2^* gene in combination with hygiene measures to keep the ToMV pressure down. According to three-dimensional structure predictions of the Tm-2^2^ and HREZ proteins (see Fig. 4E), it is likely that the *HREZ* gene acts in a similar way as the *Tm-2^2^* gene. Its effectiveness is potentially similar, suggesting that the use of the *HREZ* gene can be a durable strategy if it is widely adopted and seed companies and growers strictly apply crop hygiene procedures. The current use of IR varieties in this respect is a concern, as it allows often undetected multiplication of ToBRFV which entails the danger that more aggressive ToBRFV strains emerge.

### Conclusion

We conclude that the use of genomics tools has been indispensable in the fast discovery and introduction of high resistance to ToBRFV into tomato varieties and in understanding the working mechanism of this resistance. Knowledge about the working mechanism of genetic resistance is essential to design a durable strategy to combat this major threat to the tomato industry.

## Author Contribution Statement

WV and JRV had a clear division of responsibilities for research methodology integrity and scientific interpretation validity and prepared the manuscript, SFB, FP, RD, MY, NS, AG, WV and AJ conducted the experiments and research described herein, LL, JFT, MVS, and KK developed the HREZ varieties, KP and GJB reviewed the manuscript.

## Supplementary Material

Supplemental Tables

## Figures and Tables

**Table 1.** Assembly properties of *de novo* sequence obtained from *S. lycopersicum* plant #90479-3 before and after polishing

	Before polishing	After polishing
# Contigs	1243	1243
Total assembly size (bp)	1136562583	1136109295
Average size (bp)	914371	914006
N50 size (bp)	3125168	3124842
N50 index	89	89
N90 size (bp)	430873	431018
N90 index	464	464
# complete_busco	881	1331
% complete	61	92
# complete_and_single_copy_busco	827	1254
# complete_and_duplicated_busco	54	77
# fragmented_busco	85	32
# missing_busco	474	77
% missing	33	5
# total_busco_searched	1440	1440

**Table 2.** List of HREZ varieties introduced in 2022

Variety name	Commercial segment	Area
Arkoi	Indeterminate large beef non-heated	Mexico
Azores	Indeterminate small beef plum non-heated	South-Europe, Israel, Maghreb
Cedros	Indeterminate small beef plum non-heated	Mexico
Corsica	Indeterminate pink beef heated	Global
Haiti	Indeterminate cherry-plum heated & non-heated	Mexico
Lanzarote	Indeterminate large beef non-heated	South-Europe, Israel, Maghreb
Layeni	Indeterminate small beef round non-heated	Turkey, Middle East
Martinique	Indeterminate small beef round heated	Global
Pascua	Indeterminate cherry-plum heated & non-heated	Mexico
Pecorini	Indeterminate cherry-plum heated & non-heated	Global
Ponza	Indeterminate cherry-plum heated & non-heated	Mexico
Perimos	Indeterminate small beef round heated	Global
Shetland	Indeterminate small beef round heated	Global
Socorro	Indeterminate large beef non-heated	Mexico
Sunstream Lau	Indeterminate cherry-plum heated & non-heated	Global
Sunstream Keys	Indeterminate cherry-plum heated & non-heated	Global
Tobinaro	Indeterminate small beef round heated	Global
Ustica	Indeterminate small beef round heated	Global
