# Supplementary material for: Harnessing genomics for fast development and commercial introduction of high-resistant tomato varieties to Tomato Brown Rugose Fruit Virus
Source: Breed Sci. 2026 Feb 14;76(1):86–94. doi: 10.1270/jsbbs.25047 (PMC13437164; doi:10.1270/jsbbs.25047)
Supplement: Supplementary file 1 [file 76_086_s1.pdf]

doi: 10.1270/jsbbs.25047

**Supplemental Table 1.** Markers used for coarse mapping the ToBRFV resistance gene.

| Marker | Chromosome | Position SL2.40 |
|---|---:|---:|
| M1 | 1 | 33626858 |
| M2 | 2 | 43498502 |
| M3 | 3 | 8745728 |
| M4 | 4 | 3843807 |
| M5 | 6 | 33748837 |
| M6 | 7 | 65013231 |
| M7 | 8 | 2673609 |
| M8 | 11 | 49738156 |
| M9 | 12 | 64428284 |

**Supplemental Table 2.** Markers used for mapping the ToBRFV resistance gene on chromosome 8.

| Marker | Chromosome | position SL2.40 |
|---|---|---|
| M2 | 8 | 18072 |
| M3 | 8 | 881104 |
| M1 | 8 | 2673609 |
| M4 | 8 | 15384633 |
| M5 | 8 | 47889206 |
| M6 | 8 | 50957981 |
| M7 | 8 | 53082611 |
| M8 | 8 | 53118984 |
| M9 | 8 | 53452252 |
| M10 | 8 | 55664316 |
| M11 | 8 | 55720853 |
| M12 | 8 | 55776604 |
| M13 | 8 | 56448965 |
| M14 | 8 | 57038520 |
| M15 | 8 | 57429712 |
| M16 | 8 | 57441460 |
| M17 | 8 | 60847136 |
| M18 | 8 | 61415793 |
| M19 | 8 | 62277607 |
| M20 | 8 | 62421574 |
| M21 | 8 | 62786421 |

**Supplemental Table 3.** Markers used for fine mapping the ToBRFV resistance gene.

| Marker | Chromosome | position SL2.40 |
|---|---|---|
| M8 | 8 | 53118984 |
| M16 | 8 | 56920720 |
| M28 | 8 | 56924513 |
| M29 | 8 | 56934501 |
| M30 | 8 | 56934846 |
| M31 | 8 | 56935054 |
| M32 | 8 | 56935849 |
| M33 | 8 | 56941043 |
| M34 | 8 | 56942927 |
| M35 | 8 | 56943610 |
| M36 | 8 | 56944105 |
| M37 | 8 | 56945167 |
| M38 | 8 | 56958371 |
| M39 | 8 | 56961307 |
| M40 | 8 | 56965103 |
| M41 | 8 | 56969685 |
| M42 | 8 | 56981278 |
| M17 | 8 | 56990004 |
| M20 | 8 | 57038544 |

**Supplemental Table 4**. BlastN results (Heinz version 2.40) and description of the genes identified at the fine mapped region as well as the *HREZ* cDNA and HREZ protein sequence.

| Gene number | Best hit in SL v2.40 | Gene description |
|---|---|---|
| SEQ ID No.5 | Solyc08g075610 | Cc-nbs-lrr, resistance protein |
| SEQ ID No.6 | Solyc08g075620 | Cc-nbs-lrr, resistance protein |
| SEQ ID No.7 | Solyc08g075630 | Cc-nbs-lrr, resistance protein |
| SEQ ID No.8 | Solyc08g075640 | Cc-nbs-lrr, resistance protein |
| SEQ ID No.9 | Solyc08g075640 | Cc-nbs-lrr, resistance protein |
| SEQ ID No.10 | Solyc08g075640 | Cc-nbs-lrr, resistance protein |
| SEQ ID No.11 | Solyc08g075640 | Cc-nbs-lrr, resistance protein |
| SEQ ID No.12 | Solyc08g075600 | Receptor kinase-like protein |
| SEQ ID No.13 | Solyc08g075620 | Receptor kinase-like protein |
| SEQ ID No.14 | Solyc08g075640 | HREZ |
| SEQ ID No.15 | Solyc08g075650 | Formin 2B |
| SEQ ID No.16 | Solyc08g075670 | Formin 2B |
| SEQ ID No.17 | Solyc08g075680 | Unknown Protein |

**HREZ-cDNA**

ATGGCTGAAGCTTTCCTTCAAATTATGTTAGAGAATCTGACTTGTTTCATCCAAGGGGAAC
TTGGATTGATTCTTGGTTTTAAGGATGAGTTCGAAAAGCTTCAAAGCACGTTTACTACAAT
CCAAGCTGTGGTACAAGATGCTCAGTTGAAGCAATTGAAGGACAAGGCAATTGAAAATTG
GTTGCAGAAACTCAATGGTGCTGCATATGAAGCTGATGACATCTTGGACGAATGTAAAACT
GAGGCACCAATTATACAGAAGAAGAATAAATATGGGTGTTATCATCCAAACGTTATCACTTT
CCGTCGCAAGATTGGGAAAAGGATGAAAAAGATTATGGAGAAACTAGATGCAATTGCAGC
GGAACGAATTAAGTTTCATTTGGATGAAAGGACTATAGAGAGACAAGTTGCTACACGCCA
AACAGGTTTTGTTTTAAATGAACCACAAGTTTATGGAAGAGACAAAGATAAGGATGAGAT
AGTGAAAATCCTGATAAACAATGCCCAAACACTTTCAGTCCTCCCAATACTTGGTATGGGG
GGACTAGGAAAGACGACCCTTGCCCAAATGGTCTTCAATGATCAGAGAGTAATTGAACAT
TTCCATCCCAAAATATGGATTTGTGTCTCGGAAGATTTTAATGAAAAGAGGTTGATAAAGA
AAATTGTAGAATCTATTGAAGAAAAGTCACTTGGTGACATGGACTTGGCTCCACTTCAAAA
GAAGCTTCAGGACTTGCTGAATGGAAAAAAATATTTGCTTGTCTTAGATGATGTTTGGAAT
GAAGATCAAGATAAGTGGGCTAAGTTAAGACAAGTCTTGAAGGCTGGAGCAAGTGGTGC
TTATGTTCTAACCACTACCCGTCTTGAAAAGGTTGGATCAATCATGGGGACATTGCAACCA
TATGAATTGTCAAATTTGTCTCAAGAAGATTGTTGGTTGTTGTTCATGCAATGTGCATTTGG
GCACCAAGAAGAAATGAATCTTAATCTAGTGGCTATCGGAAAGGTGATTGTGAAAAAATG
TGGTGGTGTGCCTCTAGCAGCTAAAACTCTTGGAGGTATTTTGCGCTTCAAGAGAGAAGA
AAGACAGTGGGAACATGTGAGAGATAGTGAGATTTGGAATTTACCTCAAGATGAAAGTTC
TATTCTGCCTGCCCTGAGACTTAGTTACCATCACCTTCCACTTGATTTGAGACAATGCTTTT
CATATTGTGCAGTATTCCCAAAGGATACCAAAATGGAAAAGGAAAATCTAATCTCTCTCTG
GATGGCACATGGTTTTCTTTTATCAAAAGGAAACTTGGAGCTAGAGGATGTAGGTAATGAA
GTATGGAATGAATTATACTTGAGGTCTTTTTTCCAAGAGATTGAAGTTCAATATGATCGAAC
TTATTTCAAGATGCATGATCTCATTCATGATTTGGCAACATCTCTATTTTCAGCAAGCACATC
AAGCAGCAATATCCGAGAAATAAATGTAGAAGGTTACCTACATATGATGTCGATTGGTTTCG
CAAAAGTGGTGTCTTCTTACTCTCCTCCTCACTTGCAAAAGTTTGTCTCATTGAGGGTTCT

TAATCTAAGTTCCATGGGACTTAAGCAGTTACCGTCCTCCATTGGAGATCTAGTACATTTAA
GATACTTGAACCTCTCTCTCAATAACATGCGTACTCTTCCAAAGCAGTTATGCAAGCTTCA
AAATCTGCAGACTCTTAATGTAGAGTATTGCTGGTCACTTTGTTGTTTTCCAAAAGAAACA
AGTAAACTTGGTAGTCTCCGAAATCTCTTACTTGATGGTTGCGATGGATTGGATTCTATGCC
ACCAAGGATAGGATCTTTGACATGCCTTAAGACTCTAAGTTTATTTGTTATTGGCGAGAGA
AAAGATTCTCTACTTGGTGAATTACGAAACCTGAATCTGTATGGGTCAATTGAAATCACGC
ATCTTGAGAGAGTGAAGAATGATAGGGATGCAAAAGAAGCCAATTTATCTGCAAAAGAAA
ATCTGCATTCTTTAAGCATGAGATGGGAAGGACCACATAGATATGAATCAGAAGAAGTTGA
AGTGCTTGAATCCCTCAAACCACACTCCAATGTGACTTGTTTAACAATCACTGGCTTCAGA
GGAATCCGTCTCCCAGAGTGGATGAATCACTCAGTTTTGAAAAATGTTGTCTCTATTGCAA
TTAGAGGTTGTGAAAACTGCTCATGCTTACCACCGTTTGGTGATCTGCCTTGTCTAGAAAG
TCTAGAGTTACGGAGTGGGTCTGCGGAAGTGGAGTATGTTGAAGATTCTGGATTCCCAAC
AAGAAGAAGGTTTCCATCTATGAGAAAACTTACTATAGAAAATTTTGATAATCTGAAAGGA
TTGCTGAAAGAGGCAGGAGAAGAGCAATTCCCCGTGCTTGAAGAGTTGACAATTAGATGT
TGTCCTGTGTTTGTTATTCCGACCCTTTCTTCTGTCAAGAAATTGGTAGTTCATGGGAACAA
GTCAGATGCAATAGTTTTGAGGTCCATATATAATCTTAGGGCTCTTACTTCCCTCAACATTAG
CCATAACTTCACAGCTACTTCGCTCCCAGAAGAGATGTTCAAAAGCCTTGCAAATCTCAAA
TACTTGGAAATCGCTTTCATCTCCAATCTCAAAGAGCTGCCAAACAGCCTGGCTAGTCTCA
ATGCTTTGAAGCATCTGTTTATTAATTGTTGTTTTGCACTAGAGAGTCTCCCCGAGGAAGC
GGTGAAAGGTTTAACTTCACTCACACAGTTATCCATAACATACTGTAAGAGGCTAAAATGT
TTACCAGAGGGATTGCAGCAACTAACAAATTTATCAGTTAGGTATTGTCCAACACTGGCCA
AGCGATGTGAGAAGGGAATAGGACAAGACTGGTACAAAATTGCTCACATTCCTCATCTGC
TGATTACTGATTAG

**HREZ-protein**

MAEAFLQIMLENLTCFIQGELGLILGFKDEFEKLQSTFTTIQAVVQDAQLKQLKDKAIENWLQ
KLNGAAYEADDILDECKTEAPIIQKKNKYGCYHPNVITFRRKIGKRMKKIMEKLDAIAAERIK
FHLDERTIERQVATRQTGFVLNEPQVYGRDKDKDEIVKILINNAQTLSVLPILGMGGLGKTTL
AQMVFNDQRVIEHFHPKIWICVSEDFNEKRLIKKIVESIEEKSLGDMDLAPLQKKLQDLLNGK
KYLLVLDDVWNEDQDKWAKLRQVLKAGASGAYVLTTTRLEKVGSIMGTLQPYELSNLSQED
CWLLFMQCAFGHQEEMNLNLVAIGKVIVKKCGGVPLAAKTLGGILRFKREERQWEHVRDSEI
WNLPQDESSILPALRLSYHHLPLDLRQCFSYCAVFPKDTKMEKENLISLWMAHGFLLSKGNLE
LEDVGNEVWNELYLRSFFQEIEVQYDRTYFKMHDLIHDLATSLFSASTSSSNIREINVEGYLH
MMSIGFAKVVSSYSPPHLQKFVSLRVLNLSSMGLKQLPSSIGDLVHLRYLNLSLNNMRTLPKQ
LCKLQNLQTLNVEYCWSLCCFPKETSKLGSLRNLLLDGCDGLDSMPPRIGSLTCLKTLSLFVI
GERKDSLLGELRNLNLYGSIEITHLERVKNDRDAKEANLSAKENLHSLSMRWEGPHRYESEE
VEVLESLKPHSNVTCLTITGFRGIRLPEWMNHSVLKNVVSIAIRGCENCSCLPPFGDLPCLESL
ELRSGSAEVEYVEDSGFPTRRRFPSMRKLTIENFDNLKGLLKEAGEEQFPVLEELTIRCCPVFVI
PTLSSVKKLVVHGNKSDAIVLRSIYNLRALTSLNISHNFTATSLPEEMFKSLANLKYLEIAFISN
LKELPNSLASLNALKHLFINCCFALESLPEEAVKGLTSLTQLSITYCKRLKCLPEGLQQLTNLSV
RYCPTLAKRCEKGIGQDWYKIAHIPHLLITD

**Supplemental Table 5.** VIGS constructs used to silence the *HREZ* gene.

| VIGS construct | Sequence |
|---|---|
| ToBRFV-VIGS-01a | GGAAGATTTTAATGAAAAGAGGTTGATAAAGAAAATTGTAGAATCTATTGAAGAAAAGT<br>CACTTGGTGACATGGACTTGGCTCCACTTCAAAAGAAGCTTCAGGACTTGCTGAATGGA<br>AAAAAATATTTGCTTGTCTTAGATGATGTTTGGAATGAAGATCAAGATAAGTGGGCTAA<br>GTTAAGACAAGTCTTGAAGGCTGGAGCAAGTGGTGCTTATGTTCTAACCACTACC |
| ToBRFV-VIGS-01b | AGAAGATTTTGATGAGAAGAAGTTGATAAAGGCAATTGTTGAATCTATCGAAGGAAACC<br>CACTTGGTGACCACATGGATTTGGCTCCACTTCAAAAGAAGCTTCAGGACATGTTGAATG<br>GAAAGAGATACTTTCTCGTTTTGGATGATGTTTGGAATGAAAATCAAGAAAAGTGGGAT<br>AAGATAAAAGCAGTCTTAGAGGTTGGAGCACGAGGTGCTTCTGTTCTAACCACCACT |

**Supplemental Table 6.** Primer combination used to generate ToBRFV amplicons.

| qPCR primer name | Sequence |
|---|---|
| ToBRFV-3 Fw | ACCGTTCAACGGCAATTTAGC |
| ToBRFV-3 Rev | CCTATACACCTTAAAACCACTG |
